# Mass Spectrometry-Based Comparative Analysis of *N*‑Glycosylation Alterations in Three Human Body Fluids in Parkinson’s Disease

**DOI:** 10.1021/acschemneuro.5c00654

**Published:** 2025-10-30

**Authors:** Lingbo Zhao, Chunyan Hou, Yu Gao, Hong Jin, Chun-Feng Liu, Shuwei Li, Junfeng Ma, Shuang Yang

**Affiliations:** 1 Center for Clinical Mass Spectrometry, School of Pharmaceutical Sciences, 12582 Soochow University, Suzhou, Jiangsu 215123, China; 2 Department of Respiratory Medicine, The Fourth Affiliated Hospital of Soochow University, Suzhou, Jiangsu 215123, China; 3 Department of Oncology, Lombardi Comprehensive Cancer Center, 8368 Georgetown University Medical Center, Georgetown University, Washington, District of Columbia 20057, United States; 4 Department of Neurology and Clinical Research Center of Neurological Disease, The Second Affiliated Hospital of Soochow University, Suzhou 215004, China; 5 Nanjing Apollomics Biotech, Inc., Nanjing, Jiangsu 210033, China; 6 Laboratory of Clinical and Molecular Glycobiology, Institute of Glycomics, Shantou University Medical College, Shantou, Guangdong 515041, China

**Keywords:** Parkinson’s disease, saliva, urine, serum, glycosylation, mass spectrometry

## Abstract

Parkinson’s disease (PD) is a progressive neurodegenerative disorder lacking definitive diagnostic tests. To identify new diagnostic biomarkers, we employed glycoproteomics-mass spectrometry (MS) to investigate dynamic changes in protein *N*-glycosylation across the serum, urine, and saliva of PD patients. Our comparative analysis of differentially expressed glycoproteins (DEGs) between PD patients and healthy controls (HCs) revealed distinct patterns. Specifically, ATPase phospholipid transporter 11B (ATP11B) was significantly upregulated in the serum of PD patients, while urine and saliva showed an opposite trend. Other key findings included elevated myeloperoxidase (MPO) in urine and clusterin (CLU) in serum. Zinc-α-2-glycoprotein (AZGP1), detected in all three biofluids, displayed increased sialylation and core fucosylation in serum but decreased levels in the saliva and urine of PD patients, along with a distinct bifucosylation pattern in saliva. These glycoprotein expression changes were further validated using enzyme-linked immunoassay (ELISA). Pathway analysis indicated that these DEGs are primarily involved in inflammatory response, complement activation, and synaptic plasticity, suggesting that glycosylation dysregulation may contribute to PD progression by modulating neuroinflammation and protein homeostasis. This study represents the first comprehensive analysis of multibiofluid *N*-glycosylation in PD. The findings offer potential biomarkers and provide insights into the molecular mechanisms of the disease, which could ultimately inform early diagnosis and the development of targeted therapies.

## Graphical abstract

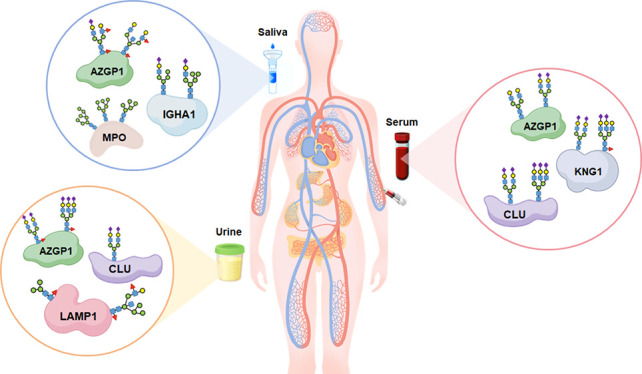

## Introduction

Parkinson’s Disease (PD) is a prevalent neurodegenerative disorder (ND) affecting approximately 10 million individuals globally, with incidence significantly increasing due to population aging, particularly affecting over 1% of individuals aged 65 and older.^1^ The pathological hallmarks of PD include the progressive loss of dopaminergic neurons in the substantia nigra and the formation of Lewy bodies, primarily composed of aggregated α-synuclein (α-Syn).^2,3^ This neuronal loss directly causes a reduction in dopamine levels, consequently leading to the characteristic motor impairments of PD.^4^ While the correlation between PD pathology and motor symptoms is established, the underlying molecular mechanisms remain incompletely understood, and neuronal loss can occur for over 20 years before a definitive pathological diagnosis is possible;^5^ furthermore, no cure exists. Consequently, early diagnosis is crucial for optimizing treatment strategies and implementing timely interventions to improve patient outcomes.

Clinical diagnosis of PD primarily depends on a neurologist’s assessment of observable motor symptoms like resting tremor, bradykinesia, rigidity, and postural instability, through a neurological examination.^6^ Although clinical diagnosis of PD is crucial, it remains subjective and challenging, particularly in early stages, due to the absence of reliable objective biomarkers and the overlap of symptoms with other conditions like dementia with Lewy bodies.^7^ Research indicates that biomarkers, especially α-Syn and blood-based markers, hold promise for early disease identification, progression tracking, and differential diagnosis.^8^ Given that PD is characterized by the loss of dopaminergic neurons and the abnormal aggregation of α-Syn in the brain, several molecular biomarkers, including α-Syn, lysosomal enzymes, neurofilaments, β-amyloid (Aβ), and Tau, have been investigated in body fluids.^9^ Despite advances in studying CSF and blood-based biomarkers, including α-Syn oligomers and neurofilament light chain (NfL), their limited sensitivity and specificity highlight the urgent need for novel molecular markers.^10,11^ Characterizing altered expression and modification of these PD-associated molecules are the key to identify the early diagnosis biomarkers.

Clinical specimens, including brain tissue, cerebrospinal fluid (CSF), peripheral blood, urine, and saliva, have been extensively utilized to identify disease-specific biomarkers. For instance, the α-synuclein seeding amplification assay (α-Syn-SAA) has been developed to detect abnormal α-Syn protein in various bodily fluids and tissue.^12^ Additionally, machine-learning approaches applied to tissue, CSF, and serum protein data have identified a reproducible multiprotein signature indicative of PD.^13^ Urinary proteomics also demonstrates promise, as urinary protein content reflects brain pathophysiological states; studies have shown significant changes in the urinary proteome in rats exposed to acute noise (119 dB),^14^ and increased levels of low molecular weight proteins and proteinuria in traumatic brain injury (TBI).^15^ Furthermore, even startle can induce dramatic alterations in urinary protein levels, affecting proteins involved in neurotransmitter transport pathways.^16^ Collectively, these findings suggest that urine represents a valuable, noninvasive source for investigating brain-associated diseases.

Given its clinical and diagnostic potential, saliva is increasingly recognized as a valuable biological fluid for biomarker discovery, potentially offering improved sensitivity.^17–19^ Its complex composition, including enzymes, proteins, cells, DNA, and RNA, allows for the detection of diverse molecular markers. Indeed, salivary transcriptomic markers (*SAT1*, *OAZ1*, *DUSP1*, etc.) and proteomic markers (IL8, IL1b) show promise for oral cancer diagnosis.^20^ Furthermore, saliva proteins reflect systemic and neurological changes, making them relevant to brain diseases; for example, preliminary studies indicate significantly elevated salivary Ab42 levels in Alzheimer’s disease (AD) compared to healthy controls (HC) (51.7 pg/mL vs 21.1 pg/mL, *p* < 0.001),^21^ and dysregulated expression of salivary total α-Syn, α-Syn oligomer/total, DJ-1, and miR-153/miR-223 in PD.^22^ These findings suggest that alterations in salivary molecules can serve as diagnostic indicators for disease onset and progression, particularly in NDs.

Protein biomarkers in human body fluids frequently undergo post-translational modifications (PTMs), including glycosylation, phosphorylation, ubiquitination, and methylation. Among these, glycosylation is prevalent on circulatory proteins and is crucial for maintaining protein conformation, mediating intercellular signaling, and providing neuroprotection.^23,24^ Emerging evidence suggests a strong correlation between aberrant glycosylation and various neurodegenerative disorders.^25,26^ For instance, α-Syn O-GlcNAcylation can modulate its aggregation and neurotoxicity,^27^ and O-GlcNAc can induce α-Syn amyloid strains with reduced seeding and pathology.^28^ Conversely, defective glycosylation of neuronal surface receptors can impair synaptic transmission and disrupt critical neuro functions, including cell signaling, axonal transport, and synaptic plasticity.^29,30^ Fortunately, these altered glycosylation patterns are detectable in peripheral body fluids, suggesting that changes in protein glycosylation in CSF and peripheral biofluids may reflect central nervous system (CNS) pathologies, offering a more convenient and less invasive alternative for early PD detection. While CSF offers a direct window into brain biochemistry and has been explored for PD diagnosis, differential diagnosis, and progression monitoring - revealing lysosomal, immune-related, and amyloid-beta subunit alterations^31,32^ - its invasive collection, limited sample availability, biomarker variability, and lack of diagnostic specificity restrict its routine clinical utility.^33^ Consequently, peripheral biofluids like serum, urine, and saliva are increasingly utilized for PD biomarker discovery. Studies have shown significant reductions in urinary N-glycan abundance, particularly biantennary galactosylated N-glycans and ST3GAL2 levels, in PD patients,^34^ and serum glycoproteomics identified increased abundance of specific N-glycans on several PD-associated proteins, including ceruloplasmin (CP), haptoglobin (HG), kininogen-1 (KNG1), complement factor H (CFH), and clusterin (CLU).^35^ These findings suggest that site-specific N-glycosylation changes in peripheral biofluids hold promise as PD biomarkers; however, the concurrent presence of these site-specific glycosylation changes across different body fluids remains unexplored.

To comprehensively identify site-specific N-glycosylation signatures in PD, we conducted a label-free nanoflow liquid chromatography-tandem mass spectrometry (nLC-MS/MS) glycoproteomic analysis of serum, urine, and saliva samples from PD patients and healthy controls (HC), as outlined in Figure 1. This workflow initiated with sample collection from both cohorts, followed by proteolytic digestion to generate peptides. Glycopeptides were then enriched using hydrophilic interaction liquid chromatography (HILIC), and N-glycans were isolated through glycoprotein enrichment for glycan analysis (GIG).^36,37^ Qualitative and quantitative analysis of glycopeptides was performed via nLC-MS/MS, while N-glycan semiquantification was achieved using MALDI-TOF-MS. Subsequently, differentially expressed glycopeptides (DEGs) were identified through comparative analysis, and their biological functions were explored using Gene Ontology (GO),^38^ Kyoto Encyclopedia of Genes and Genomes (KEGG) pathways,^39^ and protein–protein interaction (PPI) through GeneMANIA.^40^ This study seeks to reveal disease-specific glycosylation patterns in these biofluids, which could serve as potential biomarkers for early PD diagnosis and biofluid-based stratification.

**Figure 1:**
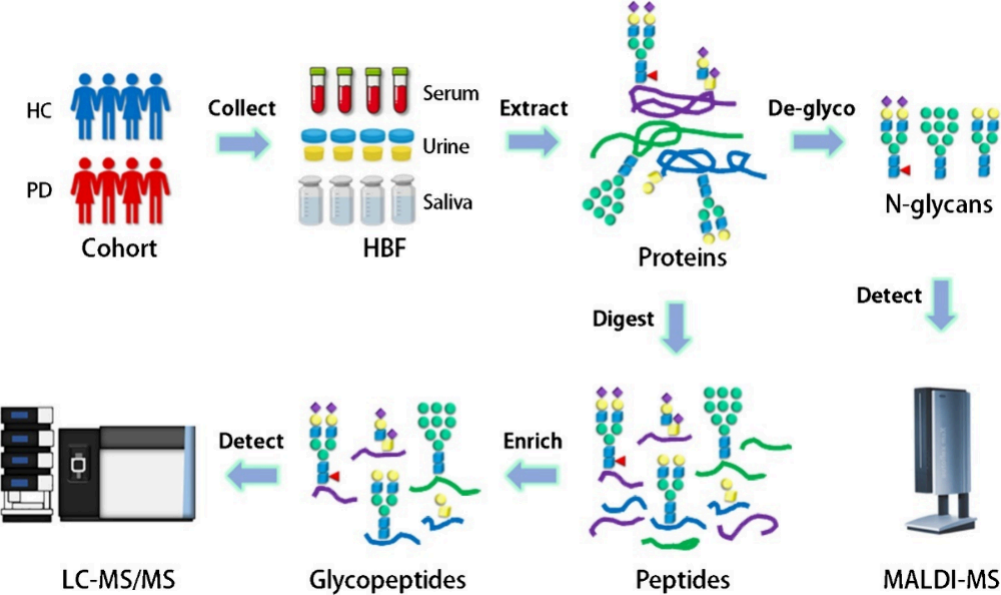
**Comprehensive workflow for glycosylation analysis of human body fluids, such as serum, urine, and saliva, using glycoproteomics-mass spectrometry.** The process begins with collecting samples from cohorts of HC and individuals with PD. Proteins are then extracted from these samples, with 10% allocated for N-glycan analysis via MALDI-TOF-MS after deglycosylation, and the remaining 90% undergoing tryptic digestion. The resulting peptides are enriched using HILIC to isolate intact glycopeptides, which are subsequently identified and quantified by LC-MS/MS. Finally, the mass spectra generated are analyzed using GlycReSoft, Byos, and flexAnalysis software to determine glycosylation patterns and identify potential biomarkers.

## Results and Discussion

### Differential N-glycan Patterns in Body Fluids

Body fluids exhibit distinct protein glycosylation patterns. Serum is characterized by a high abundance of sialoglycans, with a subset also displaying fucosylation, and a virtual absence of high-mannose glycans. In contrast, urine contains several high-mannose structures (H5N2 and H6N2), with its most prevalent N-glycan being a biantennary sialoglycan (S2H5N4) (Figure 2). Saliva presents a unique profile, featuring high-mannose glycans alongside predominantly fucosylated structures, including highly fucosylated species containing up to four fucose residues (H6N5F4). When comparing PD samples to HC, Figure 2 reveals no significant alterations in serum N-glycans, a noticeable reduction in overall N-glycan expression in PD urine, and a substantial increase in fucosylation in PD saliva. These findings suggest that urine and, particularly, saliva may offer more discriminatory glycan signatures for PD diagnosis than serum. The observed upregulation of salivary fucosylation in PD aligns with previous findings in lung adenocarcinoma, where even higher levels of fucosylation were associated with malignancy,^41,42^ reinforcing the potential of disease-specific glycan alterations in saliva as diagnostic biomarkers.

**Figure 2:**
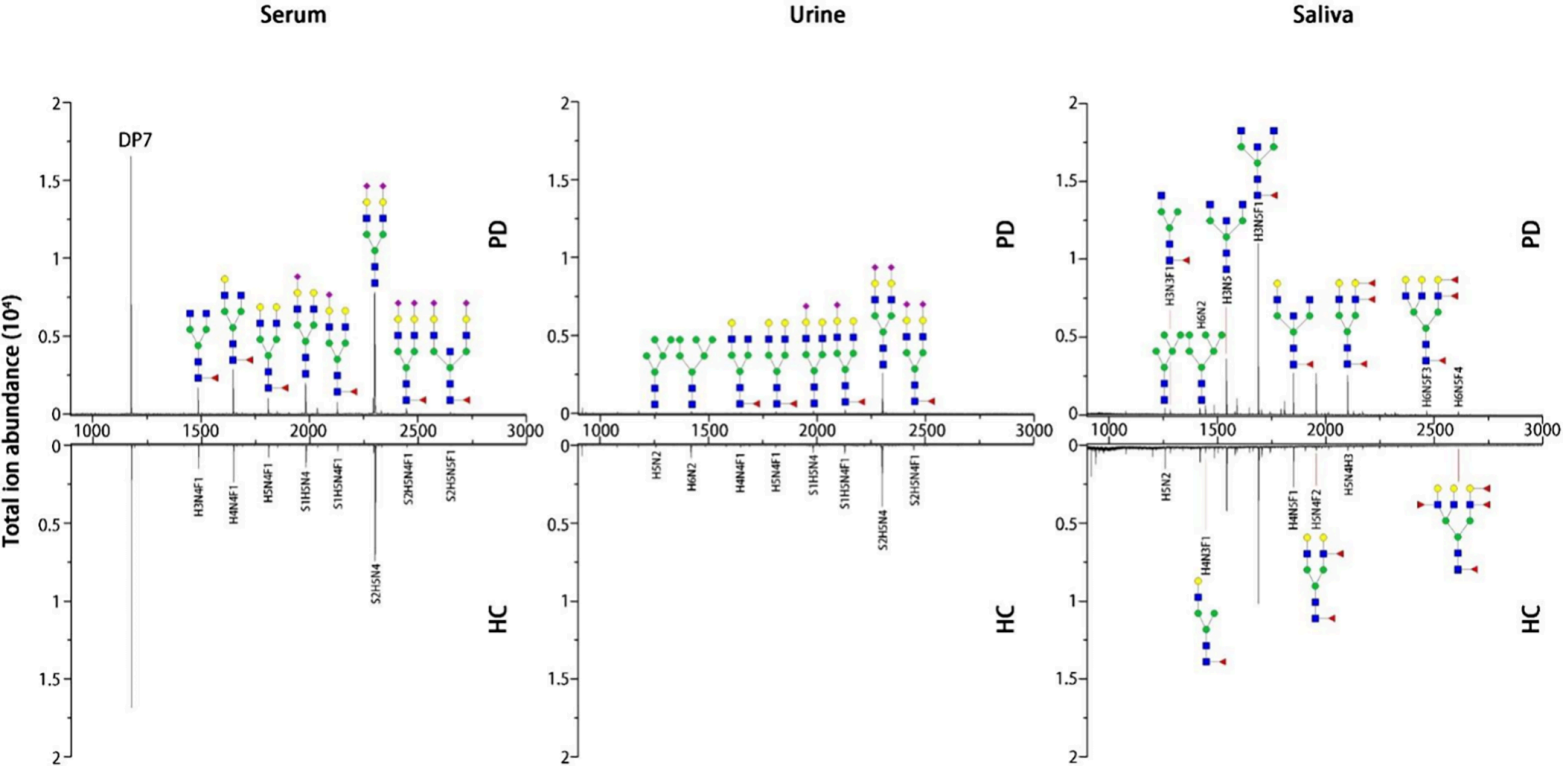
**MALDI-TOF-MS profiles of N-glycans from serum, urine, and saliva in both PD patients and HC.** Serum N-glycans, predominantly α2,6-linked sialoglycans, exhibit minimal differences between the two groups. Urine displays a variety of N-glycans including high-mannose (H5N2 and H6N2) and biantennary sialoglycans, with H6N2, S1H5N4, and S2H5N4 showing decreased abundance in PD. Saliva, in contrast, reveals a significant elevation of most N-glycan species in PD patients, including high-mannose, bisecting N-GlcNAc, and fucosylated N-glycans, suggesting its potential as a more informative biofluid for PD diagnosis compared to serum or urine. H = Hexose (Green or yellow circle), *N* = HexNAc (Blue square), S = Neu5Ac (Purple diamond), *F* = Fucose (Red triangle). Each sample was conducted in triplicate.

### PD-Specific Glycoproteins in Serum, Urine and Saliva

LC-MS analysis, as presented in Figure 3A, revealed distinct glycoprotein profiles across serum, urine, and saliva samples from PD patients compared to HC. In serum, both groups shared a substantial number of glycoproteins (88), yet PD exhibited a significantly higher number of unique glycoproteins (19) compared to HC (7), suggesting a perturbed glycoprotein landscape in PD serum. This observation was further corroborated by glycosylation site analysis, which demonstrated an increased number of unique N-glycosylation sites in PD serum (61) relative to HC serum (47, calculated as 201 total -154 shared), indicating elevated glycosylation complexity in PD. Conversely, urine analysis showed a reduction in total identified glycoproteins in PD (33) compared to HC (61), with only 5 glycoproteins unique to PD and a larger number unique to HC (33, calculated as 61 total -28 shared). This suggests a potential downregulation or altered shedding of glycoproteins in the urine of PD patients. Salivary analysis identified the fewest total glycoproteins, with 17 shared between HC and PD. However, PD saliva displayed a notable increase in unique N-glycosylation sites (22) compared to HC saliva (14), despite having fewer unique glycoproteins (4 in PD vs 7 in HC). This indicates that while the specific set of unique glycoproteins might be smaller in PD saliva, these proteins exhibit a higher degree of glycosylation complexity. Collectively, these glycoproteomic profiles across the three biofluids highlight distinct and complex alterations in glycoprotein profiles and glycosylation patterns associated with PD, suggesting that these changes may serve as potential biomarkers or reflect systemic pathological processes extending beyond the central nervous system.

**Figure 3:**
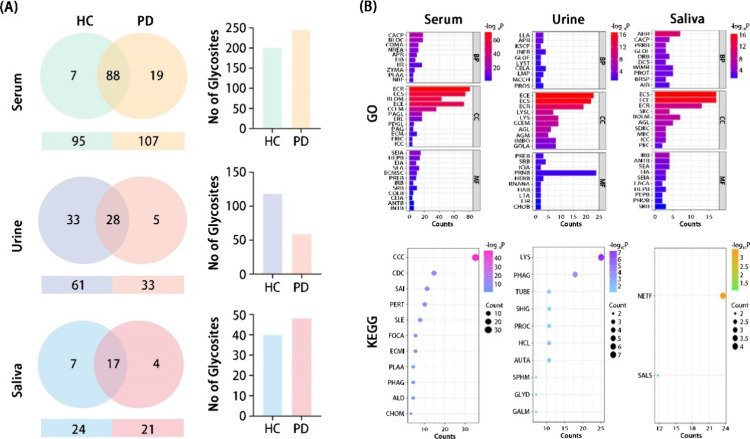
**Identification and characterization of glycoproteins in serum, urine, and saliva of PD patients.** (A) Venn diagrams reveal the overlap and unique presence of glycoproteins and glycosites across these three body fluids, demonstrating a significantly higher abundance in serum. (B) Gene Ontology (GO) analysis highlights the biological processes associated with these glycoproteins, particularly emphasizing immune and complement activation. Kyoto Encyclopedia of Genes and Genomes (KEGG) pathway analysis further elucidates the functional relevance, identifying immune-related pathways such as the complement system and coagulation cascades in serum, and neutrophil extracellular trap formation in saliva.

The proteins coidentified in PD and HC across the three body fluids were analyzed using Venn diagrams. One glycoprotein, Zinc-α-2-glycoprotein (AZGP1), was found to be coidentified in the three body fluids of both PD patients and healthy controls. AZGP1 is a key component of the perineuronal network (PNN) in the hippocampus, where it plays a role in maintaining synaptic stability, protecting neurons from oxidative stress, and regulating neural plasticity.^43^ It contributes to neuroprotection by stabilizing the extracellular environment, shielding hippocampal neurons from apoptosis, and modulating neuroinflammatory responses. As a zinc-binding protein, AZGP1 helps maintain zinc homeostasis, preventing neurotoxic accumulation of zinc and reducing oxidative stress.^44^ It also exhibits anti-inflammatory properties by inhibiting pro-inflammatory cytokine release and modulating microglial activation, which is relevant in neurodegenerative diseases like PD. Its antioxidant effects further contribute to protecting dopaminergic neurons from degeneration.^45^ Understanding the multifaceted role of AZGP1 in neuroprotection could provide new insights into therapeutic strategies aimed at mitigating neurodegenerative processes in PD and related disorders.

GO analysis across serum, urine, and saliva in PD consistently pointed to significantly impacted biological processes related to inflammation and immune response, with all three biofluids showing enrichment in terms such as immune activation and complement activation, alongside related pathways (Figure 3B, Tables S1 and S2). Moreover, the recurrent identification of extracellular space and exosomes as crucial cellular components suggests systemic PD-induced changes involving extracellular matrix remodeling and vesicle-mediated communication. Dysregulated protease activity and its regulation further indicate widespread alterations in protein breakdown and signaling across these biofluids. Supporting these findings (Table S3), KEGG pathway analysis similarly highlighted the significant involvement of immune-related pathways across all three biofluids, with specific pathways like the complement system and coagulation cascades prominent in serum, neutrophil extracellular trap formation in saliva, and lysosomal pathways in urine (more details have been discussed in Supporting Information). These results underscore the systemic nature of PD beyond neurological effects and its association with significant alterations in immune function, extracellular processes, protease activity, and lysosomal function observable across various bodily fluids.

### Alteration of Site-Specific Glycopeptides and Overall Glycoproteins

Shotgun proteomics, which analyzes constituent peptides, was employed to investigate changes in protein expression. In this study, glycopeptide intensities, quantified as the area under the curve (AUC), were summed for each glycoprotein and subsequently normalized by total ion intensity, a standard method for quantifying protein glycosylation and characterizing glycoprotein expression. It is important to note that while overall glycoprotein expression can influence glycopeptide expression, these are not always directly correlated, as glycosylation patterns and site occupancy can vary independently of total protein levels.^46,47^ We analyzed the relative abundance ratios of ten major glycoproteins in serum, urine, and saliva samples from PD patients compared to HC (Figure 4A). In serum, AFM, C4B, CFH, F9, HRG, IGHG1, KNG1, and VTN were notable, with HRG significantly increased in the HC group, and C4B and VTN elevated in the PD group. In urine, PSAP, PIGR, DSG2, UMOD, LAMP1, and AZGP1 showed significant differences, with PSAP and PIGR exhibiting higher expression in the HC group. In saliva, PRTN3, IGHG1, MUC5B, LPO, A2ML1, and ELANE were dominant, with PRTN3, IGHG1, and ELANE significantly upregulated in the PD group (Table 1). These findings highlight distinct glycoprotein expression profiles across different biological fluids, offering potential insights into the role of glycoproteins in the onset and progression of Parkinson’s disease.

**Figure 4:**
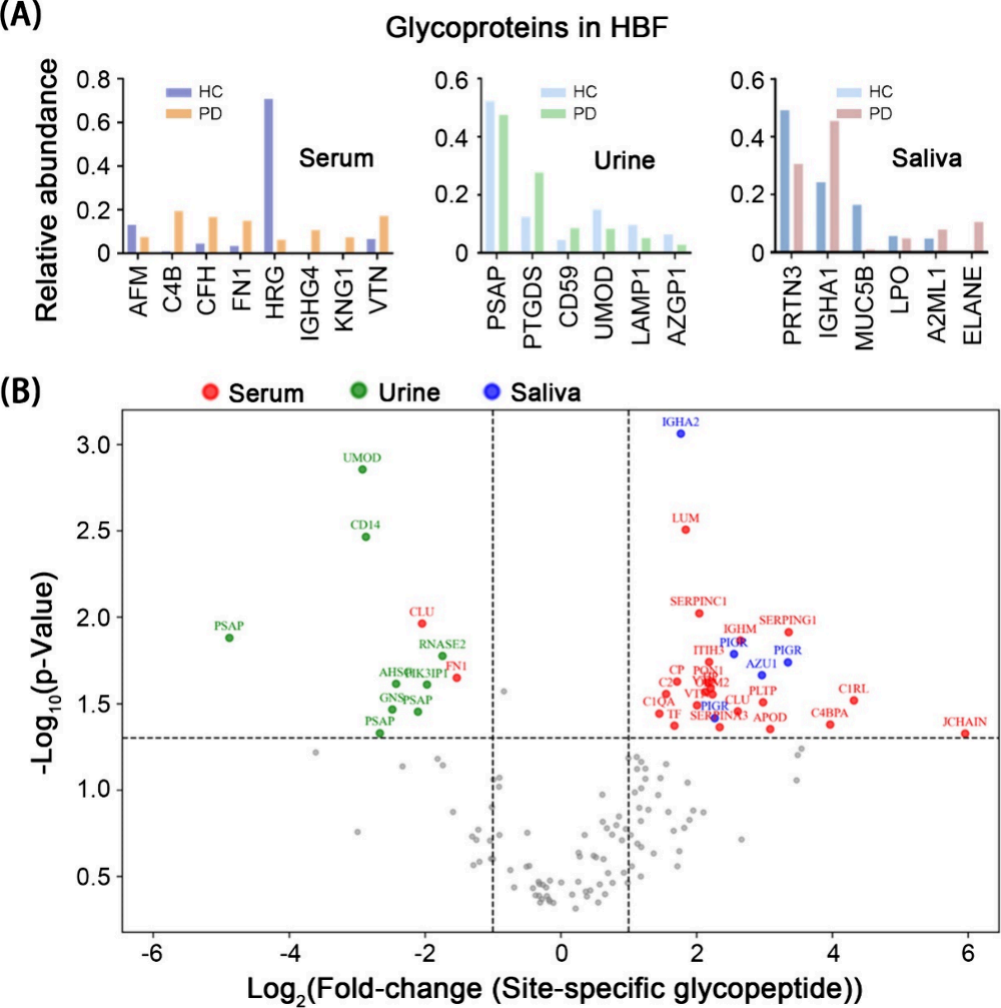
**Differential expression of site-specific glycosylation in serum, urine, and saliva of PD patients compared to HC.** (A) Relative abundance of the top 6–8 high-intensity glycoproteins across three body fluids. The intensity values of selected glycoproteins were summed and then normalized to 100% for each fluid. The *y*-axis shows the relative percentage of each glycoprotein within the total for a given fluid. The bar graphs indicate elevated expression of most glycoproteins in serum, with the exception of Afamin (AFM) and Histidine-rich glycoprotein (HRG). In contrast, urine and saliva show more heterogeneous patterns of glycoprotein expression between PD patients and HC. (B) The volcano plot illustrates significant changes, with upregulated site-specific glycopeptides in serum including LUM, SERPINC1, SERPING1, C1RL, C4BPA, APOD, TF, CP, and PON1, alongside one downregulated and one upregulated CLU glycopeptides. Conversely, urine exhibits a trend toward downregulation, notably in UMOD and PSAP, while saliva shows elevated glycopeptides such as PIGR and AZU1.

Figure 4A illustrates distinct glycoprotein expression profiles across serum, urine, and saliva, with histidine-rich glycoprotein (HRG) exhibiting the highest relative abundance in serum, prosaposin (PSAP) in urine, and proteinase 3 (PRTN3) and immunoglobulin heavy constant alpha 1 (IGHA1) in saliva. While overall glycoprotein levels may not fully capture disease-specific glycosylation alterations, quantifying intact glycopeptide levels provides a more direct measure, as the glycosylation propensity of N-glycosylation motif-containing peptides can be influenced by neighboring amino acids. To visualize glycopeptide expression differences across biofluids, volcano plots were generated, displaying the log2­(Fold change) (PD/HC) against the negative logarithm of adjusted p-values, with significance defined by |log_2_FC| > 1 and *p* < 0.05. Figure 4B and Table 2 detail the differential regulation of glycoforms (same peptide sequence with different glycans) in PD versus HC. For example, serum clusterin (CLU), also known as apolipoprotein J and implicated in PD and α-syn aggregate dynamics, shows differential regulation, with upregulation at N103 and downregulation at N86 in PD. Conversely, urine PSAP glycoforms (N80, N101, N215) are predominantly downregulated in PD, while salivary polymeric immunoglobulin receptor (PIGR) glycoforms (N186, N469, N499) are upregulated in the PD group.

**Table 1:** **Altered Fold-Change of Glycoproteins in Serum, Urine and Saliva** ^a^

	**Fold-change (FC)**		
Gene	**Serum**	**Urine**	**Saliva**
*LAMP1*	ND	0.46	ND
*ACAN*	ND	7.49	ND
*AHSG*	ND	0.4	ND
*APOD*	6.26	0.29	ND
*APOF*	ND	7.68	ND
*APOH*	2.26	ND	ND
*APOM*	0.41	ND	ND
*ATP11B*	30.1	ND	ND
*AZGP1*	ND	ND	0.34
*AZU1*	ND	14.05	2.95
*C1RL*	9.57	ND	ND
*C2*	2.14	ND	ND
*C3*	2.57	ND	0.3
*C4BPA*	11.49	ND	ND
*C4BPB*	5.04	ND	ND
*CD276*	ND	2.75	ND
*CD59*	ND	2.28	ND
*CFH*	2.43	ND	ND
*CNDP1*	2.66	ND	ND
*CTSL*	ND	4.18	ND
*ECM1*	0.38	ND	ND
*ELANE*	ND	ND	17.57
*F12*	2.44	ND	ND
*F13B*	8.8	ND	ND
*F2*	0.47	ND	ND
*FBLN1*	0.27	ND	ND
*FGB*	0.11	ND	ND
*FGG*	0.04	ND	ND
*FN1*	0.19	ND	ND
*GLB1*	ND	2.11	ND
*GRN*	5.32	ND	ND
*HGFAC*	2.04	ND	ND
*HP*	ND	ND	0.19
*IGHM*	4.55	ND	0.31
*ITIH2*	0.24	ND	ND
*ITIH3*	3.31	ND	ND
*ITIH4*	2.41	ND	ND
*ITPR3*	ND	0.18	ND
*JCHAIN*	45.26	ND	ND
*LGALS3BP*	ND	2.33	ND
*LPO*	ND	ND	0.31
*LUM*	2.61	ND	ND
*MEGF8*	2.02	ND	ND
*MPO*	ND	223.74	9.15
*MST1*	0.27	ND	ND
*MUC5B*	ND	ND	0.02
*ORM2*	3.43	ND	ND
*PLTP*	7.08	ND	ND
*PRTN3*	ND	ND	0.22
*RNASE2*	ND	3.04	ND
*SERPING1*	3	ND	ND
*TF*	ND	ND	0.09
*VWF*	0.32	ND	ND

a The ″ND″ indicates that the glycoprotein was not detected in the corresponding body fluid. The quantification for each glycoprotein was determined by summing the mass spectrometric signal of all its detected glycoforms, and this value was then normalized by the total ion abundance of all glycoforms.

**Table 2:** Differentially Expressed Site-Specific Glycopeptides in PD Compared to HC^a^

			**Site-specific N-glycans**			
**Gene**	**Protein**	**Glycosite**	**HC**	**PD**	**FC**	**HBF**
*VTN*	Vitronectin	N[86]AT	S1H5N4, S2H6N5F1	S1H5N4, S2H6N5, S2H5N4, S2H5N4F1, S3H6N5	4.0	Serum
		N[242]IS	S2H5N4F1, S1H5N4, S2H5N4, S2H6N5F1, S3H6N5F1, S3H6N5F2, S2H6N5, S3H6N5	S1H4N3, S2H5N4F1, S1H5N4, S2H5N4, S2H6N5F1, S3H6N5F1, S1H6N5, S2H6N5, S3H6N5	4.4	Serum
*SERPINA3*	a-1-antichymotrypsin	N[106]LT	S1H5N4, S2H5N4, S3H7N6, S4H7N6, S4H7N6F1	S1H5N4, S2H5N4, S2H6N5F1, S3H6N5F1, S3H6N5, S2H7N6F1, S4H7N6F1, S2H7N6, S3H7N6	5.1	Serum
*FN1*	Fibronectin	N[542]CT	H4N3, S1H4N3, H5N4, H5N4F1, S1H5N4F1, S2H5N4F1, S1H5N4, S2H5N4, S1H6N5, S2H6N5	S1H4N3, H5N4, H5N4F1, S1H5N4F1,S1H5N4, S2H5N4, S2H6N5	0.3	Serum
*PON1*	Paraoxonase 1	N[324]GT	S2H6N5, S3H6N5	S1H5N4, S2H6N5F1, S3H6N5F1, S2H6N5, S3H6N5	4.5	Serum
*C1QA*	Complement C1q subcomponent subunit A	N[146]HS	S1H5N4, S2H5N4, S2H5N4F1	S1H5N4, S2H5N4F1, H5N4F1, S1H5N4F1, S2H5N4	2.7	Serum
*C2*	Complement C2	N[621]GS	H5N2, H6N2, H7N2, H8N2, S1H6N3	H5N2, H6N2, H7N2, H8N2, S1H4N3, H6N3, S1H6N3	2.9	Serum
*LUM*	Lumican	N[127]LT	H5N4F1, S1H5N4F1,S1H6N5F1, S2H6N5F1	H5N4F1, S1H5N4F1, S2H5N4F1, H6N5F1, S1H6N5F1, S2H6N5F1, S1H7N6F1, S2H7N6F1	3.6	Serum
*SERPING1*	Plasma protease C1 inhibitor	N[253]NS	S2H5N4, S2H5N4F1, S3H6N5	S2H5N4F1, S1H5N4, S2H5N4, S2H6N5F1, S3H6N5F1, S3H6N5	10.2	Serum
*ITIH3*	Interalpha-trypsin inhibitor heavy chain H3	N[580]LT	S1H5N4, S3H6N5	S1H5N4, S2H5N4, S3H6N5F1	4.5	Serum
*ORM2*	a-1-acid glycoprotein 2	N[93]SS	S2H6N5, S4H7N6	S2H7N6, S3H7N6, S3H6N5, S2H7N6F1	4.7	Serum
*IGHM*	Immunoglobulin heavy constant mu	N[46]NS	H5N2, H6N2, S1H6N3, S1H5N3, S1H4N3, S1H5N4, H4N3F1, S1H5N5, S1H6N5, S1H6N5F1, S2H5N4F1, S2H5N5F1, H5N4, H5N4F1, H5N5F1, S1H4N3F1, S1H5N4F1	H5N2, H4N3F1, S1H4N3F1, S1H5N3F1, S1H6N3F1, S1H6N3, H4N4F1, H5N4F1, S1H5N4F1, S2H5N4F1, S1H5N4, H4N5F1, H5N5F1, S1H5N5F1, H5N5F2	6.3	Serum
*APOD*	Apolipoprotein D	N[98]LT	H5N4, S1H6N5, S1H4N3, S2H6N5F1	H4N3, H4N3F1, S1H4N3F1, S1H4N3, S1H5N3, H5N4, H5N4F1, S1H5N4F1, S1H5N4, S2H5N4, H6N5F1, S1H6N5F1, S2H6N5F1, S1H7N6F1	8.5	Serum
*PLTP*	Phospholipid transfer protein	N[64]IS	S1H6N5F1, S2H5N4F1	S1H5N4F1, S2H5N4F1, S1H5N4, H6N5F1, S1H6N5F1	7.9	Serum
*C1RL*	Complement C1r subcomponent-like protein	N[242]QT	S2H5N4, S2H5N4F1	S1H5N4, S2H5N4, S2H5N4F1	20.0	Serum
*SERPINC1*	Antithrombin-III	N[187]ET	S1H5N4	S1H5N4, S1H6N3, S2H5N4	4.1	Serum
*CP*	Ceruloplasmin	N[762]VS	S1H5N4F1, S2H5N4F1, S1H5N4, S2H5N4, S2H6N5F1, S3H6N5F1, S3H6N5F2, S1H6N5, S3H6N5	H5N4, S1H5N4F1, S1H5N4, S2H5N4, S1H6N5F1, S2H6N5F1, S2H5N4F2, S3H6N5F1, S2H5N4, S3H6N5F2, S3H6N5	3.3	Serum
*TF*	Serotransferrin	N[630]VT	S1H4N3, S1H4N4, H5N4, S1H5N4F1, S2H5N4F1, S1H5N4, S2H5N4, S2H6N5F1, S3H6N5F1, S1H6N5, S2H6N5, S3H6N5	S1H4N3, S1H4N4, H5N4, S1H5N4F1, S2H5N4F1, S2H5N4, S1H5N4, S2H6N5F1, S3H6N5F1, S3H6N5, S2H6N5, S1H6N5	3.2	Serum
*CLU*	Clusterin	N[86]ET	S2H5N4, S2H6N5F1, S3H6N5F1, S3H6N5	S3H6N5F1	0.2	Serum
		N[103]ET	S2H5N4, S3H6N5F1, S2H6N5	S2H5N4F1, S2H5N4, S2H6N5F1, S3H6N5F1, S2H6N5, S3H6N5	6.1	Serum
*C4BPA*	C4b-binding protein alpha chain	N[221]ET	S1H5N4, S2H5N4	S1H5N4, S2H5N4, S2H5N4F1, S2H6N5, S3H6N5	15.7	Serum
*JCHAIN*	Immunoglobulin J chain	N[71]IS	S1H5N3, S1H4N3F1	S1H5N3, S1H5N4F1, S2H5N4F1, S1H5N4, S1H5N5, H6N5	62.1	Serum
*AHSG*	a-2-HS-glycoprotein	N[156]DT	S2H5N4	S1H5N4, S2H5N4	0.2	Urine
*GNS*	N-acetylglucosamine-6-sulfatase	N[279]SS	H4N2, H5N2, H6N2, H7N2	H5N2, H6N2	0.2	Urine
*UMOD*	Uromodulin	N[396]ET	S1H5N4F1, S2H5N4F1, S1H5N4, S2H6N5F1, S2H6N5, S3H6N5, S2H6N6	S1H5N4, S2H6N5	0.1	Urine
*CD14*	Monocyte differentiation antigen CD14	N[282]LS	H8N2, H9N2	H9N2	0.1	Urine
*PSAP*	Prosaposin	N[80]AT	H3N2F1, S1H4N3F1, S1H5N4F1, S2H5N4F1	H3N2F1	0.0	Urine
		N[101]MS	H3N2F1, S1H4N3F1, S1H5N4F1	H3N2F1	0.2	Urine
		N[215]ST	H3N2, H3N2F1, H4N2, H5N2, H3N4F1	H3N4F1, H5N2, H3N2F1, H4N2, H3N2, S1H4N3F1	0.2	Urine
*PIK3IP1*	Phosphoinositide-3-kinase-interacting protein 1	N[66]HS	H5N4F1, S1H5N4F1, S2H5N4F1, S2H5N4F2, S2H5N4, H6N4F2, H3N5F1, H4N5F2, S2H5N5F1, S3H6N5F1	H3N5F1, S1H5N4F1, S2H5N4F1	0.3	Urine
*RNASE2*	Nonsecretory ribonuclease	N[86]MT	H3N2	H3N2	0.3	Urine
*IGHA2*	Immunoglobulin heavy constant alpha 2	N[131]LT	H5N2, H6N2, H7N2, H8N2, H9N2, H3N3, H4N3, S1H4N3, H5N3, H5N3F1, S1H5N3, S1H6N3, H3N4, H4N4, S1H4N4, H5N4, S1H5N4, S2H5N4, H3N5, H3N5F1, H4N5, S1H5N5	H3N2, H4N2, H5N2, H6N2, H7N2, H8N2, H9N2, H4N3, S1H4N3, H5N3, S1H5N3, H6N3, S1H6N3, H3N4, H3N4F1, H4N4, S1H4N4, H5N4, S1H5N4, S2H5N4, H3N5, H4N5, H4N5F1, H5N5, H5N5F1, S1H5N5, S2H5N5, S3H6N5F3	3.4	Saliva
*PIGR*	Polymeric immunoglobulin receptor	N[186]YT	H5N4F1, S1H5N4F1, S2H5N4F1	H4N3F1, S1H4N3F1, H5N4F1, S1H5N4F1, S2H5N4F1, H6N5F4	5.9	Saliva
		N[469]VT	S2H5N4, S2H5N4F2	H4N4F1, S1H4N4, H5N4F1, S1H5N4F2, S2H5N4F2, S2H5N4F3, H4N5F2, H5N5F1, H5N5F2, H5N5F3, H7N6F1	4.8	Saliva
		N[499]NT	H6N2	H5N2, H4N3, H5N4F1, S1H5N4	10.2	Saliva
*AZU1*	Azurocidin	N[171]VT	H3N2	H3N2F1, H3N2	7.8	Saliva

a The fold-change (FC) in glycopeptide expression at each N-glycosylation site was compared between the two groups. It is important to note that a single glycoprotein may show altered glycopeptide expression at multiple, distinct glycosylation sites. The FC was calculated using the relative abundance determined by mass spectrometric analysis of all glycoforms from the healthy control (HC) and Parkinson’s disease (PD) groups, respectively. The abbreviations used are as follows: H represents Hexose, N for HexNAc, F for Fucose, and S for Neu5Ac.

### Commonly Present PD-Associated Protein Glycosylation

PPI analysis was conducted to explore the functional relationships among the differentially regulated glycoproteins in human biological fluids (HBF). As illustrated in Figure 5A, the PPI networks of PD-associated glycoproteins exhibit distinct architectures across serum, urine, and saliva. The serum network displays high connectivity, with central hubs like SERPINA1 and HP, suggesting a systemic involvement of acute-phase and complement proteins in PD. The urine network, while less dense, also features SERPINA1 and HP, alongside proteins associated with renal function, potentially indicating kidney involvement or protein passage into urine. The sparse saliva network highlights localized interactions involving mucins and antimicrobial proteins, suggesting PD-related changes in oral immunity. These varying interaction landscapes across biofluids underscore the context-specific nature of PD-related protein alterations and offer potential avenues for targeted biomarker discovery and understanding the diverse pathophysiological mechanisms of the disease in different bodily compartments.

**Figure 5:**
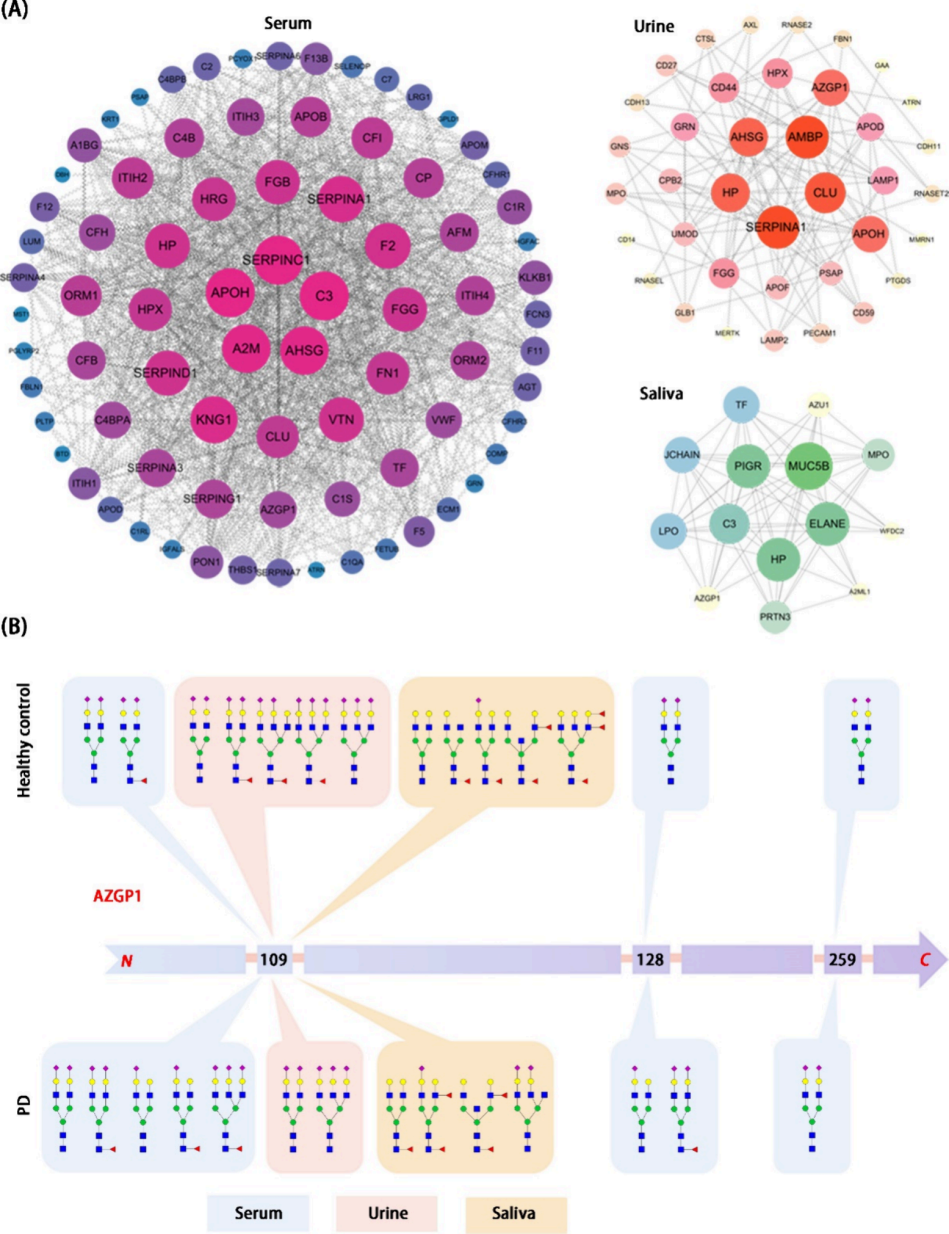
**Protein interactions and site-specific glycosylation changes in PD patients compared to HC.** (A) Protein–protein interaction (PPI) networks are presented for serum, urine, and saliva, revealing intricate connections between differentially regulated glycoproteins. The serum PPI network demonstrates a complex interplay, suggesting systemic effects beyond neurological involvement, while both urine and saliva PPI networks similarly indicate systemic changes through their respective glycoprotein interactions. (B) The schematic of AZGP1 illustrates site-specific glycosylation changes across the three body fluids. In PD serum, there’s an observed increase in the number of glycans compared to healthy controls, whereas urine and saliva show a reduction in glycan numbers at the indicated sites.

To further assess the importance of glycoproteins in different body fluids in the context of PD, PPI network information was analyzed in detail (Figure 5A). α-2-HS-glycoprotein (AHSG) and serpin family A member 1 (SERPINA1) were identified as relatively central nodes with internal correlations in both serum and urine PPI networks. AHSG, a plasma glycoprotein produced in the liver, plays a role in membrane transport.^48,49^ Takuya Kanno et al. demonstrated that AHSG is crucial for the *in vitro* cytoprotective activity mediated by WN1316, suggesting it may be an endogenous factor related to the efficacy of neuroprotective drugs.^50^ SERPINA1, a plasma serine protease inhibitor also known as α-1-antitrypsin or protease inhibitor,^51^ belongs to the serpin superfamily.^52^ It primarily forms complexes with elastase but also with plasmin and thrombin.^53^ SERPINA1 functions as an anti-inflammatory acute-phase protein by inhibiting the activation of pro-inflammatory cytokines.^54^ Distinct isoforms of SERPINA1 have been observed in the CSF of PD and PDD (PD dementia) patients in two separate studies,^55^ and elevated levels of SERPINA1 were found in the CSF of PDD and DLB (dementia with Lewy Bodies) patients compared to controls.^56^ These findings suggest that SERPINA1 could serve as a potential biomarker for the early diagnosis of dementia in PD.

Figure 5B illustrates the unique presence of AZGP1 across serum, urine, and saliva, suggesting its potential as a valuable biomarker. Consistent with previous research identifying N109, N112, N128, and N259 as potential N-glycosylation sites,^57–59^ our study detected glycosylation at N109, N128, and N259. Notably, serum AZGP1 exhibits all three glycosylation sites, while urine and saliva share a single site at N109, indicating HBF-specific glycosylation variations. Furthermore, serum from HC is characterized by predominantly biantennary sialoglycans, both with and without fucosylation, whereas PD serum displays a greater diversity of sialoglycans at N109 and N128. Conversely, urine from HC shows a wider range of sialyl and fucosyl glycans compared to PD, and saliva is characterized by multiple fucose residues. Supporting the significance of differential glycosylation, a recent study demonstrated that AZGP1 secreted by triple-negative breast cancer exhibits distinct functions compared to that secreted by ER-positive breast cancer, potentially due to variations in glycosylation.^60^ Collectively, these findings highlight the intricate and HBF-dependent nature of AZGP1 glycosylation, suggesting potential diagnostic implications, particularly for PD.

### Site-Specific PD Glycopatterns in Three Human Body Fluids

As shown in Figure S1A, several glycoproteins exhibit presence in either one or two body fluids, with the absence of others potentially attributable to their low abundance falling below MS detection limits. Concurrently, 88 glycoproteins are identified in serum, 28 in urine, and 17 in saliva across both PD and HC groups. To characterize glycopatterns, we used lectin affinity to demonstrate the presence of fucose and sialic acids (both α2,3 and α2,6 linkages) in the serum. Our results, as detailed in Table S4, show that each glycoprotein carries a specific glycopattern. Notably, CLU and MPO are observed in two body fluids. CLU shows a higher abundance in serum with six glycosylation sites, while only N354, characterized by biantennary sialoglycans, is detected in urine. Interestingly, the presence of biantennary sialic acids on CLU within urinary exosomes in PD, as previously reported,^61^ suggests sialic acids might serve as a distinct feature of PD-associated CLU.^35^ MPO, an enzyme involved in immune response through the generation of reactive oxidants, is found in both urine and saliva. Notably, MPO in PD exhibits exclusively high-mannose modifications (Man 3 to Man 7), while glycosylation at N355 is absent in HC. This indicates that N355 glycosylation may be associated with PD and potentially serve as a biomarker.

Furthermore, certain glycoproteins are uniquely identified in a single body fluid. Lysosomal associated membrane protein 1 (LAMP1) is exclusively found in urine, displaying three glycosylation sites in HC but only one at N76 in PD (Figure S1B), suggesting reduced glycosylation in PD urine. Conversely, immunoglobulin heavy constant α1 (IGHA1), found only in saliva, exhibits highly fucosylated glycans in PD. These trends highlight a pattern of reduced glycosylation in PD urine and increased fucosylation in PD saliva, compared to their HC counterparts. Complement factor H (CFH) and kininogen-1 (KNG1), both exclusive to serum, demonstrate significantly higher sialylation in PD compared to HC (Figure S1C). Overall, serum glycosylation alterations are less pronounced than those observed in urine or saliva, suggesting that urine and saliva may offer more promising avenues for the discovery of potential PD biomarkers.

### ELISA Validation of PD-Associated in Three Human Body Fluids

We used an enzyme-linked immunosorbent assay (ELISA) to independently validate the expression differences of MPO, AZGP1, and CLU in serum, saliva, and urine between PD patients and HC. This was done to provide supporting evidence at the total protein level for previous liquid chromatography-tandem mass spectrometry (LC-MS/MS) glycoproteomics findings. It is important to note that ELISA measures total protein expression and cannot distinguish between changes in glycosylation and changes in total protein. For that, more direct techniques such as lectin-based immunoassays or glycopeptide analysis are needed. Our ELISA results showed a significant and compartment-specific expression pattern for MPO: it was significantly elevated in the urine of PD patients (approximately 6-fold higher than HC, *p* < 0.0001), slightly increased in saliva (1.13-fold higher than HC, *p* < 0.05), but showed no significant difference in serum (*p* > 0.05) (Figure 6). For AZGP1, there were no significant changes in total protein abundance in serum, urine, or saliva (*p* > 0.05). Finally, for CLU, which has previously been shown to have altered sialylation in PD, our ELISA results revealed a significant upregulation in the serum of PD patients (approximately 5-fold higher than HC, *p* < 0.001), no significant difference in saliva (*p* > 0.05), and a significant downregulation in urine (*p* < 0.001) (Figure 6).

**Figure 6:**
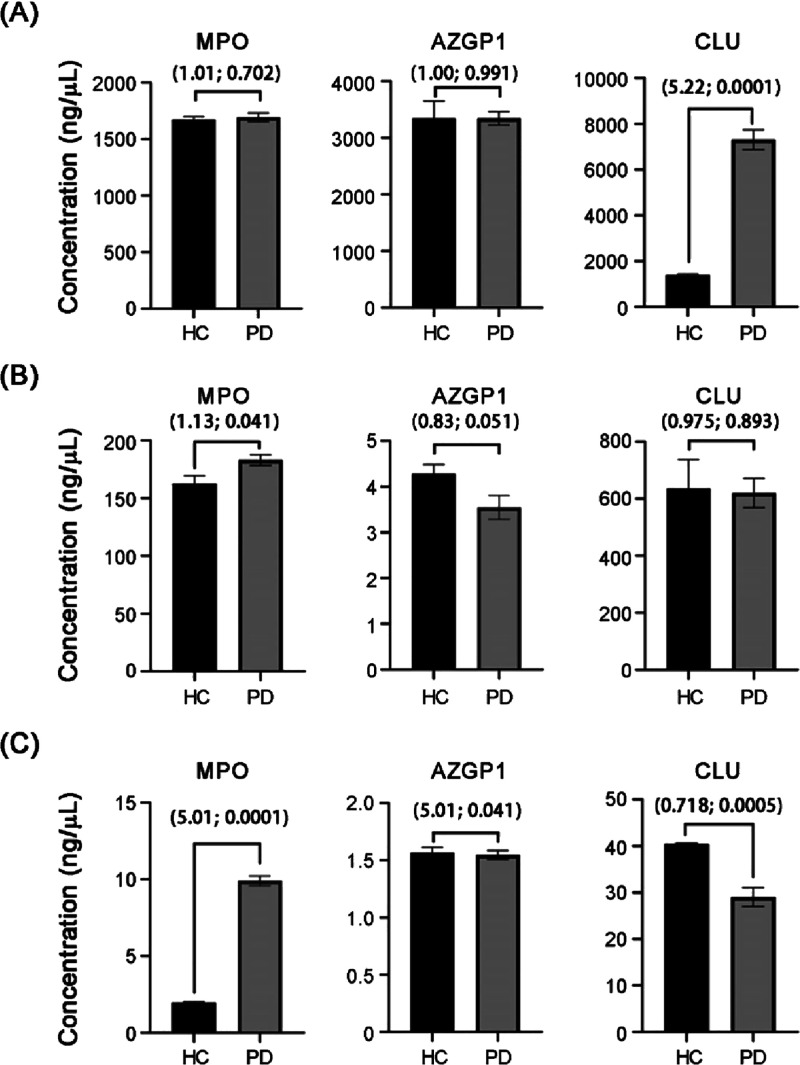
Comparison of MPO, AZGP1, and CLU protein concentrations in three body fluids from PD patients and healthy controls (HC). Protein concentrations were measured using an enzyme-linked immunosorbent assay (ELISA). (A) MPO, AZGP1, and CLU concentrations in serum. (B) MPO, AZGP1, and CLU concentrations in saliva. (C) MPO, AZGP1, and CLU concentrations in urine. The numbers in parentheses represent fold-change (FC) and the p-value from a two-tailed Student’s *t* test.

The expression trends for both MPO and CLU were consistent with our initial semiquantitative LC-MS/MS analysis. Specifically, MPO was stable in serum, slightly elevated in saliva, and significantly increased in urine, while CLU showed significant upregulation in serum, no change in saliva, and significant downregulation in urine. This high degree of consistency validates the reliability of our mass spectrometry data and provides a strong foundation for future research into the role of altered glycoprotein expression in PD. The distinct, compartment-specific expression patterns of these proteins across different biofluids suggest complex, localized pathological mechanisms in PD that require further investigation. Acknowledging the limitations of ELISA, which cannot distinguish between total protein changes and specific glycosylation alterations, future studies will incorporate glycosylation-specific detection methods to more precisely confirm and characterize the specific glycosylation changes observed in this study.

## Conclusion

This study comprehensively investigated N-glycosylation profiles across serum, urine, and saliva in PD patients using HILIC and LC-MS, revealing distinct biofluid-specific alterations. Notably, serum exhibited increased overall glycosylation site occupancy in PD, with a significant upregulation of ATP11B, a protein crucial for neuronal maturation, suggesting its potential as a PD progression biomarker. Urine showed a reduction in total glycoproteins and altered glycan expression, while saliva displayed significant fucosylation patterns, particularly in PIGR. These findings highlight the potential of urine and saliva as more discriminatory sources for PD glycan signatures compared to serum. Furthermore, the consistent identification of AZGP1 across all three biofluids, with differential glycosylation patterns, underscored its potential as a valuable biomarker and its role in neuroprotection. GO and KEGG pathway analyses revealed significant enrichment in immune-related processes, lysosomal trafficking, and MAPK signaling, correlating glycosylation dysregulation to membrane protein dysfunction and immune dys-homeostasis in PD. Collectively, this study elucidates the complex interplay between PD and biofluid-specific glycan alterations, offering novel insights into the glycobiological mechanisms underlying PD pathogenesis.

The differential N-glycan patterns observed across serum, urine, and saliva provided unique insights into potential diagnostic markers for PD. Serum primarily exhibited sialoglycans with limited fucosylation, while urine displayed a reduction in overall N-glycan expression, particularly in PD, and contained high-mannose structures. Saliva, conversely, demonstrated a substantial increase in fucosylation in PD, aligning with findings in other diseases like lung adenocarcinoma, suggesting its potential as a source of discriminatory glycan signatures. Site-specific glycopeptide analyses revealed distinct glycoprotein expression profiles across biofluids, with notable alterations in proteins like ATP11B, PIGR, and AZGP1, highlighting their roles in PD-related processes. Protein–protein interaction analysis further underscored the systemic involvement of acute-phase and complement proteins in PD, with key nodes like SERPINA1 and AHSG showing significant correlations. The observed tissue-specific glycosylation variations, particularly the increased fucosylation in saliva and altered glycosylation of AZGP1 across all biofluids, suggest that these changes reflect systemic pathological processes beyond the central nervous system and offer promising avenues for targeted biomarker discovery and understanding the diverse pathophysiological mechanisms of PD.

## Methods

### Reagents and Materials

Urea, ammonium bicarbonate (NH_4_HCO_3_), and iodoacetamide (IAA) were obtained from Aladdin Reagents (Shanghai, China), while sequencing-grade trypsin was purchased from Promega (Madison, WI, USA). Trifluoroacetic acid (TFA) and Tris (2-carboxyethyl) phosphine hydrochloride (TCEP) were acquired from Macklin (Shanghai, China), and formic acid (FA) was sourced from Sinopharm Group Chemical Reagent Company (Shanghai, China). High-performance liquid chromatography (HPLC)-grade water was obtained from J&K Chemical (Zhejiang, China), and acetonitrile (ACN) was purchased from Tedia (Fairfield, OH, USA). Spherical silica gel (C18 monomeric, 50 μm, 120 Å) was obtained from SiliCycle (Quebec, Canada), and Amide-80 gel slurry (particle size >30 μm) was purchased from Tosoh Bioscience (Tokyo, Japan). All other reagents and materials, unless otherwise specified, were procured from Beyotime (Shanghai, China).

### Sample Preparation for Glycosylation Analysis

Serum, urine, and saliva samples were collected from 40 sporadic PD patients and 30 age-matched HC (Table S5). All PD patients were diagnosed by at least two experienced neurologists according to Movement Disorders Society (MDS) clinical standards. The HC participants were recruited during routine hospital examinations and were excluded if they had a history of familial PD, Alzheimer’s disease (AD), or corticobasal degeneration. All participants provided written informed consent. The study involving human participants was approved by the Ethics Committee of the Second Affiliated Hospital of Soochow University. The institutional approval case number is JD-LK-2018–061–01.

To minimize bias, we strictly followed a standardized protocol for sample collection and processing. For serum samples, 10 μL aliquots were taken from each individual PD and HC sample to create separate pooled mixtures for each group, which were used immediately. From these pooled serum samples, 5 μL was used for glycan analysis, and 60 μL was used for intact glycopeptide analysis. For urine samples, approximately 20 mL from each participant was lyophilized. We then performed two rounds of ethanol precipitation using a 5:1 volume ratio to enrich proteins, reconstituting the dried samples in 1 mL of deionized (DI) water after each precipitation. The protein content was quantified using the Bradford BCA assay, and 500 μg of pooled protein from each group was used for intact glycoprotein analysis. For saliva samples, we first centrifuged each sample, collected the supernatant, added a protease inhibitor, and then lyophilized and redissolved the samples in DI water. For pooling, we combined 1 mL from each of 10 individual samples, resulting in four 10 mL mixtures for the PD group and three for the HC group. From each of these mixtures, a new 2 mL representative mixed sample was created. The protein concentration of these final samples was determined by the Bradford BCA assay, and 1.5 mg of total protein was used for HILIC enrichment and subsequent comprehensive glycoprotein mass spectrometry.

### HILIC Enrichment of Intact N-glycopeptides

Intact N-glycopeptides were enriched by first solubilizing samples in 100 μL of 8 M urea in 1 M ammonium bicarbonate, followed by reduction with 15 μL of 120 mM TCEP at 37 °C for 1 h. Alkylation was performed with 16.5 μL of 160 mM IAA at room temperature for 1 h in the dark. Samples were then diluted 5-fold with HPLC-grade water before overnight trypsin digestion at 37 °C with 20 μg of trypsin. Digestion was stopped by adjusting the pH to below 3 with 10% FA. Peptides were purified by solid-phase extraction (SPE) using C18 silica gel cartridges prepared with 240 μL of 50% methanol slurry. The C18 column was conditioned with three 1 mL washes of 80% ACN/0.1% TFA, followed by three 1 mL washes of 0.1% TFA. The peptide digest was loaded onto the column twice, and impurities were washed away with five 1 mL washes of 0.1% TFA. Bound peptides were eluted three times with 300 μL of 80% ACN/0.1% TFA. The eluted peptides were enriched by HILIC-SPE using Amide-80 resin.^62^ The HILIC column was conditioned with three 800 μL washes of 0.1% TFA, followed by three 800 μL washes of 80% ACN/0.1% TFA. After loading the peptides twice, the HILIC-SPE was washed three times with 80% ACN/0.1% TFA. Intact glycopeptides were eluted stepwise with 800 μL of 60% ACN/0.1% TFA, 40% ACN/0.1% TFA, and 0.1% TFA, and then concentrated to dryness. Finally, the purified N-glycopeptides were reconstituted in 0.2% FA.

### LC-MS/MS Analysis of Intact Glycopeptides

Glycopeptide analysis was performed using an Easy-nLC 1200 system coupled to a Thermo Orbitrap Fusion Lumos mass spectrometer. Approximately 1 μg of peptide sample was injected onto an EASY-Spray column (75 μm × 50 cm) packed with C18 AQ resin (2 μm, 100 Å), operating at a 250 nL/min flow rate. Chromatographic separation was achieved using a 120 min gradient of mobile phase A (0.1% formic acid in 5% acetonitrile) and mobile phase B (0.1% formic acid in 80% acetonitrile): 0.1 min at 6% B, 54 min from 6% to 22% B, 51 min from 22% to 50% B, 5 min from 50% to 60% B, 1 min from 60% to 90% B, and a 9 min hold at 90% B. Peptides were ionized via electrospray ionization at 2.2 kV. Full MS spectra were acquired from *m*/*z* 300–1600 at a resolution of 60,000 (*m*/*z* 200) with a 20 ms maximum ion accumulation time. Dynamic exclusion was enabled for 30 s. HCD MS/MS spectra were acquired at a resolution of 15,000 (*m*/*z* 200). AGC targets were set to 3 × 10^6^ for MS and 1 × 10^5^ for MS/MS. The 20 most intense precursor ions exceeding a 6.7 × 10^4^ count threshold were selected for HCD fragmentation with a 30 ms maximum ion accumulation time. A 1.2 *m*/*z* precursor isolation width was used, and singly charged and unassigned ions were excluded from MS/MS analysis. HCD was performed with a 27% normalized collision energy and a 1% underfill ratio. Data analysis was given in Supporting Information.

### GO-KEGG-PPI Analysis

We analyzed the differentially expressed glycoproteins using Gene Ontology (GO) and KEGG pathway enrichment analyses with the DAVID database (Version 6.8),^63^ setting a significance threshold of *P* < 0.05 and a false discovery rate (FDR) < 0.05. The Microbial Informatics Platform was used to visualize the enrichment results. For protein–protein interaction (PPI) analysis, we constructed a network using the STRING database (version 12.0)^64^ with a minimum interaction confidence score of 0.4. We then exported the results in TSV format to create a network diagram illustrating the relationship of these regulated glycoproteins using Cytoscape software (Version 3.9.1).^65^

### Triple Glycoprotein Level in Body Fluids by ELISA Testing

An enzyme-linked immunosorbent assay (ELISA) was used to quantify the concentrations of MPO, AZGP1, and CLU in serum, saliva, and urine samples from five healthy controls and five Parkinson’s disease patients. We used commercially available ELISA kits from Jianglai Biotechnology, specifically the Human MPO ELISA Kit (JL11580), Human AZGP1 ELISA Kit (JL22848), and Human CLU ELISA Kit (JL18979). All samples and standards were run in duplicate according to the manufacturer’s protocol, which included the following steps: standards, blanks, and diluted samples were added to the wells and incubated for 1 h at 37 °C. Next, a biotinylated antibody working solution was added and incubated for another hour, followed by three washes. An enzyme conjugate was then added, incubated for 30 min, and washed five times. Finally, TMB substrate was added and incubated in the dark for 15 min, and the reaction was stopped with a stop solution. The optical density (OD) was measured at 450 nm, and a standard curve was generated by plotting the mean absorbance values against the known standard concentrations. The concentrations of MPO, AZGP1, and CLU in the test samples were determined by extrapolating from these standard curves and multiplying by the 10x dilution factor.

## Supplementary Material



## Notes

Abbreviation (Table S1); GO analysis (Table S2); KEGG analysis (Table S3); lectin affinity (Table S4); sample information (Table S5); site-specific glycopattern (Figure S1) (PDF)
