# Supplementary material for: Mass Spectrometry-Based Comparative Analysis of N‑Glycosylation Alterations in Three Human Body Fluids in Parkinson’s Disease
Source: ACS Chem Neurosci. 2025 Oct 30;16(22):4364–76. doi: 10.1021/acschemneuro.5c00654 (PMC12636009; doi:10.1021/acschemneuro.5c00654)
Supplement: Supplementary file 1 [file cn5c00654_si_001.pdf]

Supplementary Information

Mass Spectrometry-based Comparative Analysis of N-Glycosylation Alterations in Three Human Body Fluids in Parkinson's Disease

Lingbo Zhao[1,2$], Chunyan Hou[3$], Yu Gao[1,2$], Hong Jin[4], Chun-Feng Liu[4], Shuwei Li[5], Junfeng Ma[3*], Shuang Yang[1,2,6*]

[1] Center for Clinical Mass Spectrometry, School of Pharmaceutical Sciences, Soochow University, Jiangsu 215123, China

[2] Department of Respiratory Medicine, The Fourth Affiliated Hospital of Soochow University, Suzhou, Jiangsu 215123, China

[3] Department of Oncology, Lombardi Comprehensive Cancer Center, Georgetown University Medical Center, Georgetown University, Washington, DC 20057, USA

[4] Department of Neurology and Clinical Research Center of Neurological Disease, The Second Affiliated Hospital of Soochow University, Suzhou, 215004, China

[5] Nanjing Apollomics Biotech, Inc., Nanjing, Jiangsu 210033, China

[6] Laboratory of Clinical and Molecular Glycobiology, Institute of Glycomics, Shantou University Medical College, Shantou, Guangdong 515041, China.

[$] These authors contributed equally to this work.

*Please address all inquiries to the corresponding authors, Dr. Shuang Yang, shuangyang@stu.edu.cn, Shantou University Medical College; Dr. Junfeng Ma, junfeng.ma@georgetown.edu, Georgetown University.

Table of Contents

Supporting Information - Methods 3

MALDI-TOF-MS 3

LC-MS/MS data analysis 4

Intact glycopeptide volcano plot 4

Supporting Information - Results and Discussion 4

GO and KEGG pathway enrichment 4

Supplementary Tables and Figures 7

Table S1. Abbreviation of terminology of Gene Ontology (GO) and Kyoto Encyclopedia of Genes and Genomes 7

Table S2. Gene oncology (GO) enrichment of biological processes (BP), cellular components (CC) and molecular functions (MF) found in glycoproteomic analysis of serum, urine and saliva. 10

Table S3. Summary of key and shared KEGG pathways associated with glycoproteins identified in three types of biological fluids. 11

Table S4. Lectin affinity identification of serum glycoproteins in PD patients. 12

Table S5. Clinical characteristics of specimens (saliva, serum and urine) collected from patients and healthy controls. 15

Figure S1. Site-specific glycopeptide variations in PD patients compared to HC across different body fluids. 19

Reference 20

Supporting Information - Methods

MALDI-TOF-MS

Glycoproteins were prepared for analysis using a solid-phase chemoenzymatic method to release N-glycans. First, 200 μL of AminoLink Plus resin was added to an empty spin column and washed with 500 μL of 1× coupling buffer twice. Protein samples (1 mg each) were then diluted to 475 μL with 1× coupling buffer and incubated with the resin for four hours at room temperature to facilitate protein coupling. Next, 25 μL of 1 M $NaBH_3CN$ was added, and the mixture was incubated for over four hours on a rotary mixer. The coupling buffer was replaced with 1× PBS, and the samples were incubated for another four hours with 50 mM $NaBH_3CN$. To block any remaining aldehyde groups, 1 M Tris-HCl (pH 7.4) was added. Sialic acids were derivatized with 0.25 M EDC/0.25 M HOBt hydrate in ethanol for one hour at 37°C, followed by labeling with 1 M p-Toluidine (pT). The resin was then washed sequentially with 500 μL of 10% formic acid (three times), 10% acetonitrile (three times), 1 M NaCl (three times), and deionized water (three times). Finally, 1 μL of PNGase F was added to 200 μL of 25 mM NH_4HCO_3 and incubated at 37°C for at least four hours or overnight to release the N-glycans.

For glycan quantification, maltoheptaose (DP7) was used as an internal standard at a final concentration of 20 μM. One microliter of the N-glycan solution was spotted onto a MALDI 384 target plate and air-dried. Following this, 1 μL of matrix solution (50 mg/mL DHB in 50% ACN, 0.1 mM NaCl, and 2% DMA) was applied and air-dried. A Bruker ultrafleXtreme MALDI-MS was used for N-glycan detection in positive ion mode with a mass range of 900-4000 Da. The laser intensity was set between 40-60%, with a frequency of 2000 Hz and 1000 laser pulses per shot. To ensure data quality, the signal-to-noise (S/N) ratio for N-glycan mass spectra was set to 2, and areas with high signal intensity and stable peaks were selected for detection. Signals were accumulated three times per spot, and each sample was measured in at least four independent replicates to ensure accuracy and consistency.

LC-MS/MS data analysis

Analysis of the mass spectrometry data began by converting the 'psm.tsv' output file from MSFragger into an .xlsx format. To ensure high-confidence data, we filtered the peptide segments based on a 'Glycan q-value' of less than 0.05, an 'Intensity' greater than 0, and 'Is unique' set to TRUE. After removing duplicate entries, the VLOOKUP function was used to retrieve the corresponding proteins for these filtered peptides. We then performed a relative quantitative analysis on glycoproteins co-identified in the serum, saliva, and urine of Parkinson's disease (PD) patients and healthy controls (HC) to screen for differentially expressed glycoproteins. The identified differential glycoproteins were further analyzed using bioinformatics methods, including Protein-Protein Interaction (PPI), Gene Ontology (GO), and Kyoto Encyclopedia of Genes and Genomes (KEGG) analyses. Additionally, we examined the different glycosylation sites and glycoforms of the same glycoprotein across the three biofluids. Statistical analysis of the glycosylation changes was performed using GraphPad Prism 9.0.0 software.

Intact glycopeptide volcano plot

We analyzed the data by first selecting peptides that met the criteria of 'Glycan q-value' < 0.05, 'Intensity' > 0, and 'Is unique' = TRUE. Duplicate entries were then removed to ensure high-confidence data. Using the VLOOKUP function, these filtered peptides were mapped to their corresponding proteins. For each biofluid, we retained peptides that were detected more than three times for the analysis of differentially expressed proteins. The abundance of each peptide was determined by its detection count, which was then multiplied by the 'Intensity' value to calculate a theoretical intensity for p-value computation. Peptides with a p-value < 0.05 and a $|log2FC| > 1$ were considered statistically significant and were used to generate a volcano plot.

Supporting Information - Results and Discussion

GO and KEGG pathway enrichment

GO and KEGG pathway enrichment analyses provided valuable insights into the biological functions of differentially expressed proteins and their involvement in the complex pathogenesis

of PD. By leveraging these bioinformatics tools, we identified several critical biological functions and pathways likely implicated in the molecular mechanisms underlying PD. Notably, inflammation emerged as a central player in PD pathology, where sustained neuroinflammatory processes are linked to the progressive degeneration of dopaminergic neurons [1]. Chronic neuroinflammation, often mediated by activated microglia and astrocytes, contributes to an environment that fosters oxidative stress, mitochondrial dysfunction, and protein misfolding—factors closely associated with PD progression [2]. Complement system activation represents another significant pathway identified in our analyses. Aberrant activation of complement proteins can potentiate neuroinflammation and lead to synaptic pruning, ultimately resulting in the loss of dopaminergic neurons in the substantia nigra, a hallmark of PD [3]. Furthermore, the dysregulation of proteolytic enzymes, such as serine proteases, has been implicated in the modulation of inflammatory cascades [4]. The inhibition of these proteases may play a neuroprotective role by attenuating inflammatory responses and promoting neuronal survival, suggesting potential therapeutic targets for mitigating neurodegeneration in PD [5].

In addition to inflammatory pathways, protein aggregation and impaired proteostasis are key features observed in PD. Misfolded proteins, such as a-synuclein, tend to aggregate and form Lewy bodies, which are toxic to neurons and disrupt normal cellular processes [6]. The convergence of these pathogenic pathways, including immune responses, oxidative stress, and proteostasis imbalance [7], underscores the multifactorial nature of PD. **Table S2** provides a detailed summary of the primary and overlapping GO terms associated with glycoproteins identified in serum, urine, and saliva. These glycoproteins may serve as potential biomarkers for early diagnosis and monitoring of PD progression. Collectively, our findings highlight that multiple-interconnected biological pathways—including inflammation, complement activation, protein aggregation, and immune responses—may play critical roles in the etiology and progression of PD. Understanding these complex interactions could pave the way for novel therapeutic strategies aimed at halting or reversing the progression of PD.

Table S3 summarizes the KEGG pathways identified in serum, urine, and saliva samples from PD patients. These pathways include phagosome and complement-coagulation cascades, both of which play significant roles in the pathophysiology of PD [8]. The phagosome pathway is involved in the phagocytosis and degradation of waste materials and pathogens and is mediated by signaling pathways such as PI3K/Akt and MAPK [9]. Dysregulation of this pathway can lead to the accumulation of protein aggregates that cannot be effectively cleared, exacerbating neuronal damage. The complement and coagulation cascades pathway, associated with the complement and coagulation systems, is crucial for infection defense and tissue repair [10]. Overactivation of this pathway can result in chronic inflammatory responses, microvascular complications, and neuroinflammation in PD patients [11]. The cited references in **Table S3** provide supporting evidence for these associations, underscoring the potential critical roles these pathways may play in the onset and progression of PD.

Supplementary Tables and Figures

Table S1. Abbreviation of terminology of Gene Ontology (GO) and Kyoto Encyclopedia of Genes and Genomes.

Abbreviation	Full name
CACP	Complement activation, classical pathway
BLOC	Blood coagulation
COMA	Complement activation
NREA	Negative regulation of endopeptidase activity
APR	Acute-phase response
FIB	Fibrinolysis
IIR	Innate immune response
ZYMA	Zymogen activation
PLAA	Plasminogen activation
NRF	Negative regulation of fibrinolysis
ECR	Extracellular region
ECS	Extracellular space
BLOM	Blood microparticle
ECE	Extracellular exosome
CCEM	Collagen-containing extracellular matrix
PAGL	Platelet alpha granule lumen
ERL	Endoplasmic reticulum lumen
PDGL	Platelet dense granule lumen
PAG	Platelet alpha granule
ECM	Extracellular matrix
FIBC	Fibrinolysis complex
ICC	Immunoglobulin complex, circulating
SEIA	Serine-type endopeptidase inhibitor activity
HEPB	Heparin binding
EIA	Endopeptidase inhibitor activity
SEA	Serine-type endopeptidase activity
ECMSC	Extracellular matrix structural constituent
PREB	Protease binding
IRB	Immunoglobulin receptor binding
SRB	Signaling receptor binding
COLB	Collagen binding
CEIA	Cysteine-type endopeptidase inhibitor activity
ANTB	Antigen binding
INTB	Integrin binding
LLA	Lysosomal lumen acidification
KSCP	Keratin sulfate catabolic process
INFR	Inflammatory response
GLOF	Glomerular filtration

LYST	Lysosomal transport
CELA	Cell adhesion
LMP	Lipid metabolic process
MCCH	Muscle cell cellular homeostasis
PROS	Protein stabilization
LYSL	Lysosomal lumen
LYS	Lysosome
AGL	Azurophil granule lumen
AGM	Azurophil granule membrane
IMBO	Intracellular membrane-bounded organelle
GOLA	Golgi apparatus
ICIA	Ion channel inhibitor activity
PREB	Protease binding
PRNB	Protein binding
RNANA	RNA nuclease activity
HAB	Hyaluronic acid binding
LTA	Lipid transporter activity
CHOB	Cholesterol binding
AHR	Antibacterial humoral response
PRRB	Positive regulation of respiratory burst
DRB	Defense response to bacterium
DCS	Detection of chemical stimulus involved in sensory perception of bitter taste
IMMR	Immune response
PROT	Proteolysis
BRSP	B-cell receptor signaling pathway
AIR	Adaptive immune response
SIIC	Secretory IgA immunoglobulin complex
SDIIC	Secretory dimeric IgA immunoglobulin complex
MIIC	Monomeric IgA immunoglobulin complex
PIIC	Pentameric IgM immunoglobulin complex
LACA	Lactoperoxidase activity
PEPB	Peptidoglycan binding
PHOB	Phosphatidylcholine binding
CCC	Complement and coagulation cascades
CDC	Coronavirus disease - COVID 19
SAI	Staphylococcus aureus infection
PERT	Pertussis
SLE	Systemic lupus erythematosus
FOCA	Focal adhesion
ECMI	ECM-receptor interaction
PLAA	Platelet activation
PHAG	Phagosome
ALD	Alcoholic liver disease
CHOM	Cholesterol metabolism

TUBE	Tuberculosis
SHIG	Shigellosis
PROC	Proteoglycans in cancer
HCL	Hematopoietic cell lineage
AUTA	Autophagy - animal
SPHM	Sphingolipid metabolism
GLYD	Glycosaminoglycan degradation
GALM	Galactose metabolism
NETF	Neutrophil extracellular trap formation
SALS	Salivary secretion

Table S2. Gene oncology (GO) enrichment of biological processes (BP), cellular components (CC) and molecular functions (MF) found in glycoproteomic analysis of serum, urine and saliva. The key biological activities involved in these fluids were revealed.

Category	Term	Correlation to PD	Ref
BP	Acute-phase response	Inflammation and immune response dysregulation are associated with neurodegeneration in PD.	[Ayton S, et al. ***Mov Disord.*** 2022]
BP	Complement activation (classical pathway)	Complement activation contributes to neuroinflammation and dopaminergic neuron loss in PD.	[Loeffler DA, et al. ***J Neuroinflammation.*** 2006]
BP	Inflammatory response	Chronic inflammation is a known risk factor for PD progression.	[Stojkovska I, et al. ***Exp Biol Med.*** 2015]
BP	Fibrinolysis	Altered fibrinolysis is linked to cerebrovascular abnormalities and cognitive decline in PD patients.	[Reuland CJ, et al. ***Med Hypotheses***. 2020]
BP	Innate immune response	Dysfunctional innate immunity plays a role in PD pathogenesis.	[Harms AS, et al. ***Acta Neuropathol.*** 2021]
CC	Extracellular space	Extracellular proteins are involved in PD-related neurodegenerative processes.	[EL Andaloussi S, er al. ***Nat Rev Drug Discov.*** 2013]
CC	Extracellular exosomes	Involved in cell communication, potentially related to the spread of pathological proteins	[Pinnell JR, et al. ***J Neurochem.*** 2021]
MF	Serine-type endopeptidase inhibitor activity	Inhibition of protease activity can impact inflammatory cascades and neuronal survival in PD.	[Jaako K, et al. ***J Cell Sci.*** 2016]
MF	Protein binding	Aberrant protein-protein interactions, including α-synuclein aggregation, are central to PD pathology.	[Di Maio R, et al. ***Sci Transl Med.*** 2016]
MF	Antigen binding	Dysregulated immune surveillance and antigen processing may contribute to PD progression.	[Hobson BD, et al. ***J Parkinsons Dis.*** 2022]

Table S3. Summary of key and shared KEGG pathways associated with glycoproteins identified in three types of biological fluids.

Pathway	Function	Signaling Pathways	Association with Parkinson's Disease	Ref
Phagosome	Phagocytosis and degradation of waste, pathogens, etc.	PI3K/Akt, MAPK	Dysfunction leads to the accumulation of protein aggregates that cannot be cleared, exacerbating neuronal damage	[Portugal, et al. The FEBS Journal. 2022] [Tan Y, et al. International journal of biological macromolecules. 2020]
Complement and coagulation cascades	Defense against infection and tissue repair	Complement system, coagulation system	Overactivation leads to chronic inflammatory response, microvascular disease, and neuroinflammation	[Ma S X, et al. Journal of proteome research. 2021] [Naskar A, et al. ACS chemical neuroscience. 2022]

Table S4. Lectin affinity identification of serum glycoproteins in PD patients. The Uniprot # column lists the specific ID for each identified glycoprotein. The lectins AAL, SNA, and MAL-II were used to identify specific glycan structures: fucose, α2,6-linked sialic acid, and α2,3-linked sialic acid, respectively.

Uniprot #	Protein	Fucose	α2,6	α2,3
A0AUZ9	KAT8 regulatory NSL complex subunit 1-like protein	√		
O75443	α-tectorin	√		
O94916	Nuclear factor of activated T-cells 5	√		
O95497	Pantetheinase			√
P00450	Ceruloplasmin	√	√	√
P00734	Prothrombin		√	√
P00738	Haptoglobin	√	√	√
P00739	Haptoglobin-related protein	√	√	
P01008	Antithrombin-III		√	√
P01009	α-1-antitrypsin	√	√	√
P01011	α-1-antichymotrypsin	√	√	√
P01023	α-2-macroglobulin	√	√	√
P01024	Complement C3	√	√	√
P01031	Complement C5		√	√
P01042	Kininogen-1		√	√
P01860	Immunoglobulin heavy constant γ3	√	√	√
P01861	Immunoglobulin heavy constant γ4	√	√	√
P01871	Immunoglobulin heavy constant mu	√	√	√
P01876	Immunoglobulin heavy constant α 1		√	√
P01877	Immunoglobulin heavy constant α 2		√	
P02671	Fibrinogen α chain		√	√
P02748	Complement component C9			√
P02749	β-2-glycoprotein 1		√	√
P02751	Fibronectin		√	√
P02760	Protein AMBP			√
P02763	α-1-acid glycoprotein 1		√	√
P02765	α-2-HS-glycoprotein			√
P02766	Transthyretin	√	√	√
P02787	Serotransferrin	√	√	√
P02790	Hemopexin		√	√
P03952	Plasma kallikrein			√
P04003	C4b-binding protein α chain			√
P04004	Vitronectin		√	
P04114	Apolipoprotein B-100		√	√
P05154	Plasma serine protease inhibitor		√	
P05155	Plasma protease C1 inhibitor		√	√

P05546	Heparin cofactor 2		√	√
P07333	Macrophage colony-stimulating factor 1 receptor	√		
P07357	Complement component C8 α chain			√
P08603	Complement factor H	√	√	√
P09871	Complement C1s subcomponent			√
P10643	Complement component C7			√
P10909	Clusterin	√	√	√
P13725	Oncostatin-M			√
P19652	α-1-acid glycoprotein 2		√	√
P19823	Inter-alpha-trypsin inhibitor heavy chain H2		√	√
P19827	Inter-alpha-trypsin inhibitor heavy chain H1		√	√
P22792	Carboxypeptidase N subunit 2			√
P22891	Vitamin K-dependent protein Z			√
P23142	Fibulin-1		√	
P25311	Zinc-α-2-glycoprotein			√
P27169	Serum paraoxonase/arylesterase 1			√
P35125	Ubiquitin carboxyl-terminal hydrolase 6	√		
P35916	Vascular endothelial growth factor receptor 3		√	
P49746	Thrombospondin-3	√		
P82094	TATA element modulatory factor		√	
P98164	Low-density lipoprotein receptor-related protein 2		√	
Q02224	Centromere-associated protein E	√		
Q03591	Complement factor H-related protein 1			√
Q13029	PR domain zinc finger protein 2		√	
Q15303	Receptor tyrosine-protein kinase erbB-4		√	
Q2KJY2	Kinesin-like protein KIF26B			√
Q494X3	Zinc finger protein 404		√	
Q52LW3	Rho GTPase-activating protein 29		√	
Q5T1H1	Protein eyes shut homolog			√
Q5VV63	Attractin-like protein 1	√	√	
Q6N041	Uncharacterized protein DKFZp686O16217		√	
Q6N091	Uncharacterized protein DKFZp686C02220		√	
Q6S5L8	SHC-transforming protein 4			√
Q86TH1	ADAMTS-like protein 2		√	
Q8N139	ATP-binding cassette sub-family A member 6		√	√
Q8N6G6	ADAMTS-like protein 1		√	√
Q8NDA2	Hemicentin-2	√		
Q96MP5	Zinc finger SWIM domain-containing protein 3		√	
Q96PD5	N-acetylmuramoyl-L-alanine amidase		√	√
Q96RV3	Pecanex-like protein 1		√	
Q9BSJ5	Uncharacterized protein C17orf80	√		
Q9NRF2	SH2B adapter protein 1		√	
Q9NYQ6	Cadherin EGF LAG seven-pass G-type receptor 1	√		

Q9NZR2	Low-density lipoprotein receptor-related protein 1B	√	
Q9P2N2	Rho GTPase-activating protein 28		√
Q9UK05	Growth/differentiation factor 2	√	

Table S5. Clinical characteristics of specimens (saliva, serum and urine) collected from patients and healthy controls. Abbreviations: PD#, number of Parkinson's disease patients; HC#, number of healthy control individuals; M, male; F, female; LEDD, L-dopa equivalent daily dose; H-Y, Hoehn and Yahr scale.

**Sample #**	**Age**	**Gender**	**Disease duration** (Year)	**Drug therapy** (LEDD)	**H-Y**
PD1	63	M	2	0	1
PD2	40	F	1	188	1.5
PD3	74	M	2	300	1.5
PD4	43	M	2	0	1
PD5	58	M	10	975	1
PD6	70	F	2	300	1.5
PD7	35	M	2	225	1.5
PD8	70	F	3	375	3
PD9	66	F	1.5	0	1
PD10	65	M	3	400	2
PD11	61	M	1	188	2
PD12	44	F	3	300	1
PD13	77	M	8	450	2
PD14	57	M	0.5	300	1.5
PD15	47	M	3	300	2
PD16	71	M	3	375	2
PD17	53	M	2	225	2
PD18	66	F	1.5	0	1
PD19	47	M	3	300	2
PD20	65	M	3	400	2
PD21	61	M	1	188	2
PD22	44	F	3	300	1
PD23	70	F	3	375	3
PD24	77	M	8	450	2
PD25	57	M	0.5	300	1.5

Sample #	Age	Gender	Disease duration (Year)	Drug therapy (LEDD)	H-Y
PD26	71	M	3	375	2
PD27	53	M	2	225	2
PD28	43	M	1	300	1
PD29	51	F	3	275	2
PD30	65	M	3	225	2
PD31	46	F	2	188	1.5
PD32	76	M	4	225	1
PD33	49	M	1	300	3
PD34	59	M	2	300	2
PD35	58	M	2.5	400	1
PD36	66	M	3	275	1.5
PD37	61	F	1.5	450	1.5
PD38	57	F	0.5	375	2
PD39	69	M	2	300	2
PD40	70	F	2	225	2
HC1	62	M	/	/	/
HC2	52	F	/	/	/
HC3	54	F	/	/	/
HC4	58	F	/	/	/
HC5	60	M	/	/	/
HC6	56	F	/	/	/
HC7	65	M	/	/	/
HC8	59	M	/	/	/
HC9	66	M	/	/	/
HC10	57	M	/	/	/
HC11	60	F	/	/	/
HC12	52	F	/	/	/
HC13	54	F	/	/	/
HC14	53	M	/	/	/
HC15	61	M	/	/	/
HC16	68	M	/	/	/

Sample #	Age	Gender	Disease duration (Year)	Drug therapy (LEDD)	H-Y
HC17	51	F	/	/	/
HC18	60	F	/	/	/
HC19	52	F	/	/	/
HC20	55	F	/	/	/
HC21	57	F	/	/	/
HC22	50	F	/	/	/
HC23	51	F	/	/	/
HC24	65	M	/	/	/
HC25	55	M	/	/	/
HC26	46	F	/	/	/
HC27	51	F	/	/	/
HC28	71	F	/	/	/
HC29	65	F	/	/	/
HC30	56	M	/	/	/

Figure S1. Site-specific glycopeptide variations in PD patients compared to HC across different body fluids. (A) It presents glycopeptides identified in two body fluids, specifically CLU (Clusterin) found in both serum and urine, and MPO (Myeloperoxidase) found in saliva and urine, showcasing differential glycosylation patterns between PD and HC. (B) The figure highlights glycopeptides unique to a single body fluid, such as LAMP1 (Lysosomal-associated membrane protein 1) exclusively in urine and IGHA1 (Immunoglobulin heavy constant alpha 1) exclusively in saliva. (C) Finally, it illustrates glycopeptides detected only in serum, focusing on two key PD-associated glycoproteins, CFH (Complement factor H) and KNG1 (Kininogen-1), demonstrating distinct glycosylation profiles between PD and HC within this fluid.

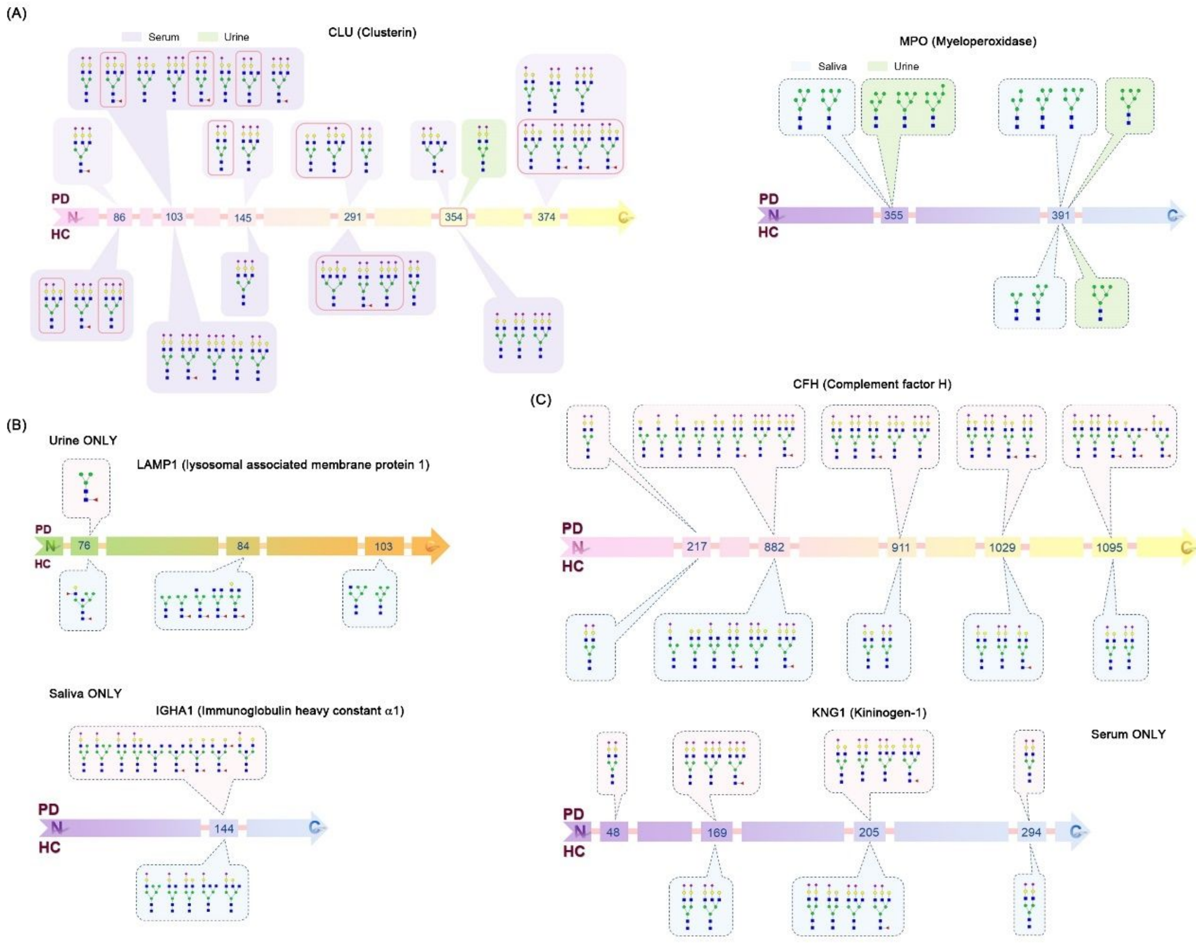

Reference

(1) Zhang, W.; Xiao, D.; Mao, Q.; Xia, H. Role of neuroinflammation in neurodegeneration development, *Sig. Transduct. Target Ther.* **2023**, *8*, 1-32.

(2) Dash, U. C.; Bhol, N. K.; Swain, S. K.; Samal, R. R.; Nayak, P. K.; Raina, V.; Panda, S. K.; Kerry, R. G.; Duttaroy, A. K.; Jena, A. B. Oxidative stress and inflammation in the pathogenesis of neurological disorders: Mechanisms and implications, *Acta Pharm. Sin. B.* **2025**, *15*, 15-34.

(3) Fatoba, O.; Itokazu, T.; Yamashita, T. Microglia as therapeutic target in central nervous system disorders, *J. Pharmacol. Sci.* **2020**, *144*, 102-118.

(4) Sharony, R.; Yu, P.-J.; Park, J.; Galloway, A. C.; Mignatti, P.; Pintucci, G. Protein targets of inflammatory serine proteases and cardiovascular disease, *J. Inflamm.* **2010**, *7*, 1-17.

(5) Kip, E.; Parr-Brownlie, L. C. Reducing neuroinflammation via therapeutic compounds and lifestyle to prevent or delay progression of Parkinson’s disease, *Ageing Res. Rev.* **2022**, *78*, 1-23.

(6) Stefanis, L. α-Synuclein in Parkinson's Disease, *Cold Spring Harb. Perspect. Med.* **2012**, *2*.

(7) Dick, F.; Tysnes, O.-B.; Alves, G. W.; Nido, G. S.; Tzoulis, C. Altered transcriptome-proteome coupling indicates aberrant proteostasis in Parkinson’s disease, *iScience* **2023**, *26*, 1-20.

(8) Tremblay, M.-E.; Cookson, M. R.; Civiero, L. Glial phagocytic clearance in Parkinson’s disease, *Mol. Neurodegeneration* **2019**, *14*, 1-14.

(9) Goyal, A.; Agrawal, A.; Verma, A.; Dubey, N. The PI3K-AKT pathway: A plausible therapeutic target in Parkinson's disease, *Exp. Mol. Pathol.* **2023**, *129*, 1-10.

(10) Satyam, A.; Graef, E. R.; Lapchak, P. H.; Tsokos, M. G.; Dalle Lucca, J. J.; Tsokos, G. C. Complement and coagulation cascades in trauma, *Acute Med. Surg.* **2019**, *6*, 329-335.

(11) Foley, J. H.; Conway, E. M. Cross talk pathways between coagulation and inflammation, *Circ. Res.* **2016**, *118*, 1392-1408.
